# COVID-19-Associated Pulmonary Aspergillosis Complicated by Severe Coronavirus Disease: Is Detection of Aspergillus in Airway Specimens Before Disease Onset an Indicator of Antifungal Prophylaxis?

**DOI:** 10.7759/cureus.36212

**Published:** 2023-03-16

**Authors:** Takaaki Kitayama, Kazuya Tone, Koichi Makimura, Masamichi Takagi, Kazuyoshi Kuwano

**Affiliations:** 1 Department of Respiratory Medicine, The Jikei University School of Medicine Kashiwa Hospital, Chiba, JPN; 2 Division of Mycosis Control, Teikyo University Institute of Medical Mycology, Tokyo, JPN; 3 Division of Respiratory Diseases, Department of Internal Medicine, The Jikei University School of Medicine, Tokyo, JPN

**Keywords:** covid-19-associated pulmonary aspergillosis, aspergillus fumigatus, antifungal prophylaxis, tocilizumab, corticosteroid, coronavirus disease

## Abstract

A 55-year-old man was admitted for coronavirus disease 2019 (COVID-19)-related respiratory failure. He was treated with corticosteroids and tocilizumab in the intensive care unit. *Aspergillus* *fumigatus (A. fumigatus)* was isolated from his sputum on admission. However, no radiological findings suggesting pulmonary aspergillosis were seen on chest computed tomography (CT). Since the fungus had merely colonized in airways, antifungal drugs were not administered immediately. On day 19 of hospitalization, a high (1→3)-β-D-glucan (BDG) level was noted. A CT scan on day 22 revealed consolidations with a cavity in the right lung. *A. fumigatus* was isolated from his sputum again. Thus, we diagnosed the patient with COVID-19-associated pulmonary aspergillosis (CAPA) and started voriconazole. After the treatment, BDG levels and radiological findings were noted to improve. In this case, tocilizumab probably had a critical role in developing the disease. Although antifungal prophylaxis therapy for CAPA is not well established, this case shows that detecting *Aspergillus* in airway specimens before the disease onset possibly implies a high risk of developing CAPA and is an indicator of antifungal prophylaxis.

## Introduction

Patients with coronavirus disease 2019 (COVID-19), similar to those with severe influenza, could develop acute respiratory distress syndrome (ARDS) [1]; therefore, the emerging coronavirus is called severe acute respiratory syndrome coronavirus 2 (SARS-CoV2). Severe COVID-19 is treated with corticosteroids [2], immunosuppressive agents [3], and antiviral drugs in the intensive care unit (ICU). These conditions may lead to secondary pulmonary aspergillosis, reported as COVID-19-associated pulmonary aspergillosis (CAPA). The mortality rate is extremely high in invasive pulmonary aspergillosis (IPA), the most severe manifestation of aspergillosis [4]. CAPA is believed to be one type of IPA; thus, early detection and treatment are deemed desirable [5]. Here, we report a patient with CAPA who benefited from early antifungal treatment and discuss antifungal prophylaxis for the disease.

## Case presentation

A 55-year-old man with type 2 diabetes, hypertension, dyslipidemia, and sleep apnea syndrome presented with pyrexia, malaise, and dyspnea on exertion five days before admission. One day before admission, he had a polymerase chain reaction test for SARS-CoV2 at another hospital and was diagnosed with COVID-19. He had been a smoker for 40 years, smoking approximately 40 cigarettes per day.

**Figure 1:**
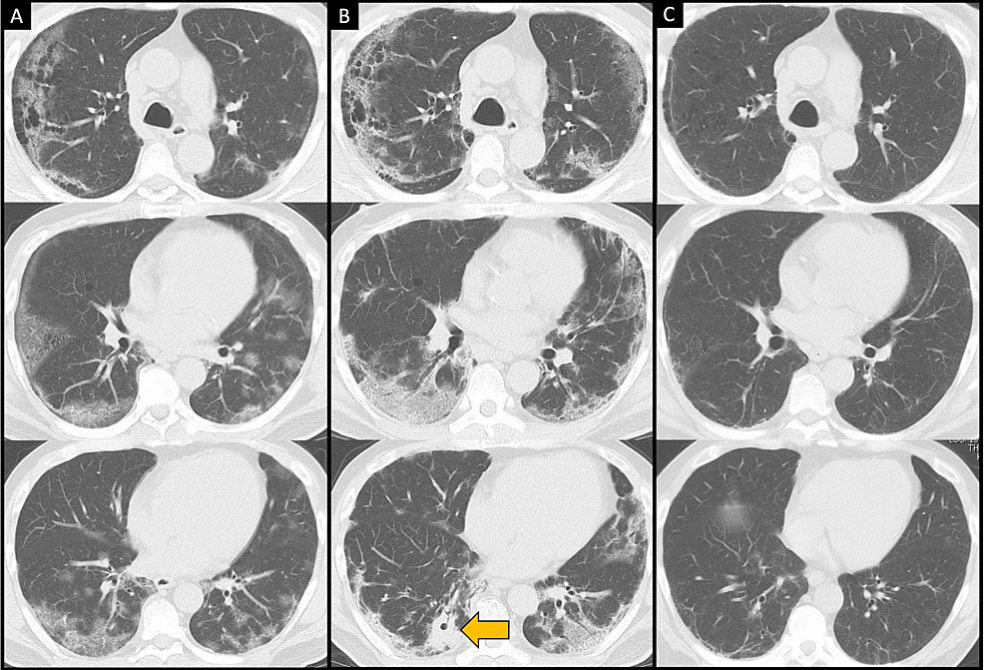
Chest computed tomography findings A) On admission, showing bilateral diffuse ground-glass opacities dominantly on the peripheral side with emphysema B) At the onset of pulmonary aspergillosis, showing consolidations with a cavity (arrow) C) After the antifungal treatment for 12 weeks, showing that the *Aspergillus* cavitary lesion with consolidation, as well as bilateral ground-glass opacities of SARS-CoV-2 pneumonia, improved

**Figure 2:**
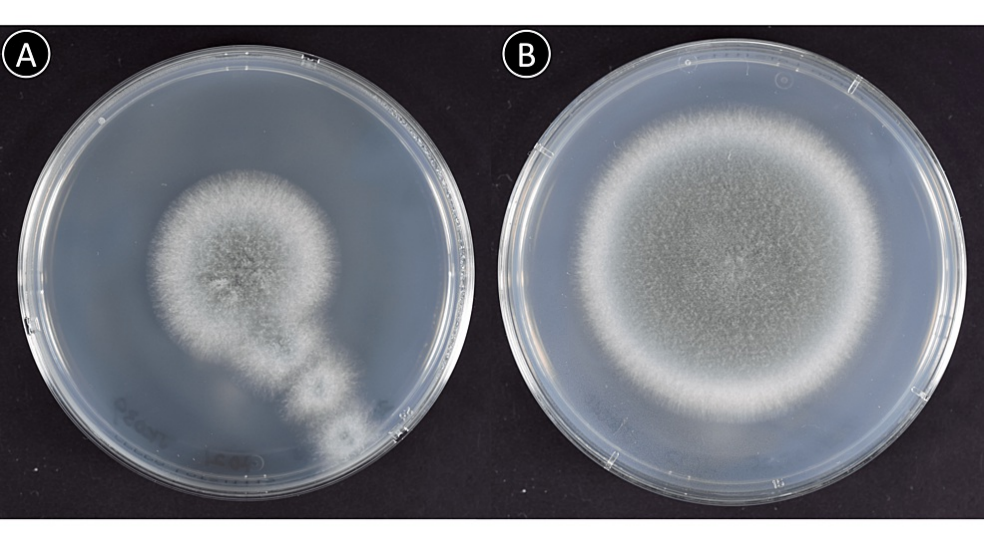
Giant colony findings of the clinical isolates on admission (A, JK 039) and at the onset of pulmonary aspergillosis (B, JK 040)

Physical examination on admission revealed a temperature of 39.4°C, pulse rate of 104 beats/min, blood pressure of 154/73 mmHg, respiratory rate of 20 breaths/min, and peripheral oxygen saturation of 90% with ambient air. Blood tests revealed increased inflammation (C-reactive protein 14.90 mg/dL (reference value 0.00-0.14 mg/dL) and ferritin 911 ng/mL (reference value 14-304 ng/mL)), elevated lactate dehydrogenase (379 U/L (reference value 124-222 U/L)), abnormal coagulation parameters (D-dimer 1.6 μg/mL), renal dysfunction (creatinine 2.99 mg/dL), and poorly controlled diabetes (fasting blood sugar 208 mg/dL and hemoglobin A1c 8.4%). His (1→3)-β-D-glucan (BDG) level was normal. Computed tomography (CT) revealed pulmonary emphysema and ground-glass opacities in the peripheral lung (Fig 1A). Although *Aspergillus sp*. was isolated from sputum on admission (Fig 2A, strain JK039), no radiological findings suggestive of pulmonary aspergillosis on chest CT were seen (Fig 1A). Therefore, the treatment was started with dexamethasone (6.6 mg/day) and anticoagulation therapy with heparin calcium (10,000 units/day) but without antifungal agents. Because of renal dysfunction, the antiviral drug remdesivir was not administered. On day three of hospitalization, he was admitted to the ICU due to a deterioration in his respiratory condition that necessitated high-flow nasal cannula oxygen therapy. He was then prescribed intravenous corticosteroid pulse therapy with methylprednisolone at 500 mg/day for three days and a dose of tocilizumab at 8 mg/kg. On day six of hospitalization, he was prescribed dexamethasone again at 13.2 mg/day. The patient recovered from pyrexia, and his respiratory condition gradually improved. After withdrawing nasal high-flow oxygen therapy on day seven, the patient was transferred from the ICU to the general ward on day 12. Dexamethasone was tapered off from day 16 and was completely discontinued on day 23 of admission.

**Figure 3:**
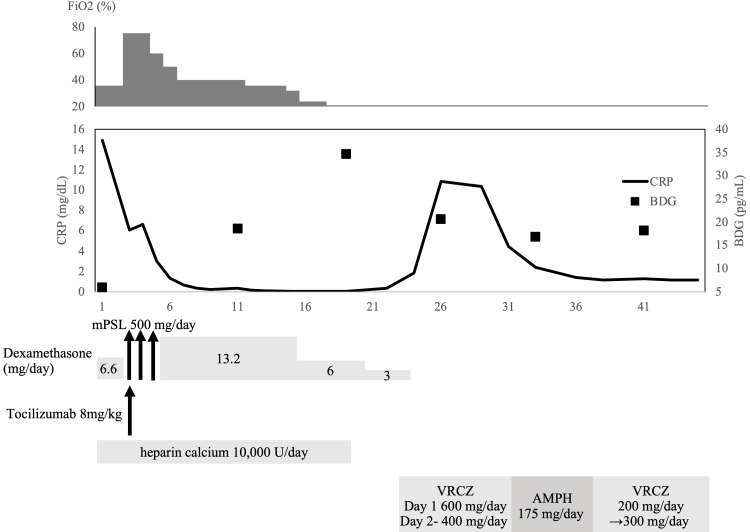
Clinical course CRP, C-reactive protein; BDG, (1→3)-β-D-glucan; mPSL, methylprednisolone; VRCZ, voriconazole; L-AMB, liposomal amphotericin B.

However, a high BDG level (30.7 pg/mL, cutoff value 11.0 pg/mL, Fujifilm Wako BDG assay) was found on day 19 (Fig 3), and a CT scan performed on day 22 revealed consolidations with a cavity in the right lower lobe (Fig 1B, arrow). Although the serum *Aspergillus* galactomannan antigen test was negative, considering the presence of *Aspergillus* in previous sputum cultures, we clinically diagnosed the patient with CAPA. We started oral voriconazole (600 mg/day as loading and then 400 mg/day). C-reactive protein and BDG levels improved after starting voriconazole. Although *Aspergillus sp.* was also isolated from sputum collected on day 22 immediately before the antifungal treatment (Fig 2B, strain JK040), the fungus was no longer detected after the treatment. Since the voriconazole-induced hepatotoxicity emerged, we switched to intravenous liposomal amphotericin B (175 mg/day, approximately 3mg/kg [6]), and the liver damage improved. In anticipation of continued treatment after discharge from the hospital, we switched to a reduced dose of voriconazole (200 mg/day) with ursodeoxycholic acid on day 37. The patient was discharged on day 45. Antifungal treatment was given for 12 weeks. After discharge from the hospital, the consolidations continued to shrink on CT examination (Fig 1C).

Microbiological analysis

The total DNAs of two clinical isolates of *Aspergillus sp.* were extracted using the ethanol-chloroform method. The beta-tubulin partial sequences of BT1, BT2 [7], and BenA [8] regions were amplified. Purified amplicons were directly sequenced, and the identities were 100% matches for *A. fumigatus* on the BLAST database (https://blast.ncbi.nlm.nih.gov/Blast.cgi). To confirm that the two isolates were of the exact origin, we conducted multilocus sequencing typing (MLST) using ANXC4, BGT1, CAT1, LIP, MATI-2, SODB, and ZRF2 primers [9]. The sequence types are shown in Table 1. PubMLST revealed that the two clinical isolates had different origins (https://pubmlst.org/organisms/*A. fumigatus*). Table 2 shows the drug susceptibility test results on the two clinical isolates.

**Table 1:** Multilocus sequencing typing of clinical isolates from sputum This table shows each sequence type for clinical isolates of *Aspergillus fumigatus* (JK 039 and JK 040).

Clinical isolates (Strain)	ANX4	MAT1_2	SODB	BGT1	ZRF2	LIP	CAT1	Sequence type
*A. fumigatus* (JK 039)	2	2	1	1	1	2	2	24
*A. fumigatus* (JK 040)	1	2	1	1	1	2	2	7

**Table 2:** Antifungal susceptibility tests according to CLSI M38-A2 for clinical isolates from sputum MCFG, micafungin; CPFG, caspofungin; AMPH, amphotericin B; 5-FC, flucytosine; FLCZ, fluconazole; ITCZ, itraconazole; VRCZ, voriconazole; MCZ, miconazole For MCFG and CPFG, the minimal effective concentration (μg/mL) was determined. Moreover, the minimum inhibitory concentration  (μg/mL)  was measured for other drugs.

Clinical isolates (Strain)	MCFG	CPFG	AMPH	5-FC	FLCZ	ITCZ	VRCZ
*A. fumigatus* (JK 039)	≤0.015	1	2	>64	>64	0.5	0.25
*A. fumigatus* (JK 040)	≤0.015	0.5	1	>64	>64	0.5	0.25

## Discussion

COVID-19 is still a worldwide pandemic, even though it has been over three years since its first reported outbreak. In critically ill patients, secondary infections are often observed during intensive care or immunosuppressive therapies for severe pneumonia brought on by excessive immune responses [10].

The present case is valuable because it suggests two critical points. First, CAPA is a significant complication of COVID-19, and early diagnosis and treatment may improve patient prognosis. Second, antifungal prophylaxis for the prevention of CAPA may be considered for COVID-19 patients whose cultures are positive for *Aspergillus* before the onset of CAPA with a high aspergillosis risk.

Meersseman et al. [11] retrospectively analyzed data and found that 6.9% (127) of the 1850 ICU admissions had microbiological or histopathologic evidence of aspergillosis. Patients with advanced acquired immunodeficiency syndrome, prolonged neutropenia, allogeneic hematopoietic stem cell transplant, solid organ transplant, preexisting lung disease, and long-term use of corticosteroids or other immunosuppressive agents are at risk of developing invasive aspergillosis [12]. The clinical course of COVID-19 is similar to that of severe influenza infection, causing lymphocytopenia and ARDS associated with cytokine storm [1]. COVID-19 patients may be susceptible to IPA because acute respiratory failure caused by influenza is a risk factor for IPA [13]. Additionally, several cohort studies have shown that relatively high doses or prolonged treatment with corticosteroids (more than several weeks) may be a risk factor for CAPA [14,15]. In COVID-19 patients with ARDS, tocilizumab, an IL-6 receptor antagonist, significantly improves clinical outcomes [16]. However, the use of tocilizumab may paradoxically increase the predisposition to CAPA. Recent European Confederation of Medical Mycology (ECMM) research found that CAPA was a strong independent predictor of ICU mortality and was more common in older patients receiving invasive ventilation and tocilizumab [17]. However, unexpectedly, underlying factors, such as smoking, diabetes mellitus, pulmonary diseases, or glucocorticoid therapy, are not CAPA risk factors, although they are univariable predictors of worse 90-day-ICU outcomes [6]. Therefore, we concluded that tocilizumab was critical in developing CAPA in this case.

Another critical point is that *Aspergillus* was detected in his sputum culture early in his treatment. In practice, CAPA has been categorized using various definitions. The ECMM and the International Society for Human and Animal Mycology published consensus criteria for identifying CAPA in December 2020, classifying patients as having proven, probable, or possible CAPA [6]. According to a systematic review of CAPA [18], the diagnosis was made using lower respiratory tract culture in 77.6% (149/192) of CAPA patients, with bronchoalveolar lavage fluid (BALF) being the primary source. Fungal biomarkers galactomannan antigen (GM) and BDG have been reported in CAPA diagnosis. Approximately 18.2% (35/192) of CAPA patients were GM positive in serum, while 45.3% (87/192) were positive in BALF when the cutoff values were defined as 0.5 for serum and 1.0 for BALF. Other serum biomarkers of BDG were positive for only 10.4% (20/192) of patients diagnosed with CAPA. In the same review, the overall mortality rate for patients diagnosed with CAPA was 48.4% (93/192), with variations in mortality among hospitals. Antifungal therapy should commence in patients suspected of IPA as soon as possible because diagnostic testing is limited, and early treatment may slow the disease progression [19]. No study has directly compared the effectiveness of antifungal drugs for CAPA; thus, IPA treatment is recommended [6]. For possible, probable, and proven CAPA, voriconazole or isavuconazole can be used as a first-line treatment option [6]. The optimal duration of antifungal therapy for CAPA is unknown; however, an expert panel recommends a 6-12-week treatment period [6]. Although BALF could not be investigated from the viewpoint of infection control, GM in serum was negative, BDG was positive, a new consolidation with a cavity emerged on CT, and *Aspergillus* was detected in sputum culture. Therefore, we diagnosed possible CAPA based on the ECMM criteria and started treatment with voriconazole immediately, leading to a good outcome.

Additionally, although *A. fumigatus* was isolated before the onset of CAPA, the role of antifungal prophylaxis in CAPA remains unclear. Hatzl et al. reported that antifungal prophylaxis does not improve the outcome but reduces the risk of developing CAPA [20]. However, this report also mentions that COVID-19 patients with CAPA had a poor outcomes; therefore, the efficacy of antifungal prophylaxis for the prevention of CAPA is not concluded. As this report is a single-center non-randomized observational study, a multicenter randomized trial will be required to determine the significance of antifungal prophylaxis in severe COVID-19.

Detecting *Aspergillus* in airway specimens before the onset of CAPA, as in this case, has not been examined for antifungal prophylaxis. In the present case, the two strains of *A. fumigatus* isolated before and after the onset were found to be genetically different from MLST, although drug susceptibilities were similar; hence, it cannot be concluded that antifungal prophylaxis had to be performed. Nevertheless, lung airway epithelial cells damaged by COVID-19 or smoking favor easy colonization and growth of the fungus. Thus, if *Aspergillus* is isolated from airway specimens before the disease onset in patients with severe COVID-19 who have risk factors such as tocilizumab or corticosteroid use, as well as emphysema, it may be better to consider antifungal prophylaxis. Moreover, the novelty in the present case lies in the discussion of the need for antifungal prophylaxis based on MLST results of clinical isolates before and after the onset of CAPA.

## Conclusions

We report a patient diagnosed with pulmonary aspergillosis while receiving COVID-19 treatment and had a good outcome using antifungal agents. In patients with CAPA, it may be necessary to identify CAPA risk factors and perform early diagnosis and treatment, sometimes prophylactically, to achieve a favorable outcome.

## References

[1] Armstrong-James D, Youngs J, Bicanic T (2020). Confronting and mitigating the risk of COVID-19 associated pulmonary aspergillosis. Eur Respir J.

[2] Horby P, Lim WS, Emberson JR (2021). Dexamethasone in hospitalized patients with Covid-19. N Engl J Med.

[3] Toniati P, Piva S, Cattalini M (2020). Tocilizumab for the treatment of severe COVID-19 pneumonia with hyperinflammatory syndrome and acute respiratory failure: a single center study of 100 patients in Brescia, Italy. Autoimmun Rev.

[4] Kosmidis C, Denning DW (2015). The clinical spectrum of pulmonary aspergillosis. Thorax.

[5] Koehler P, Cornely OA, Böttiger BW (2020). COVID-19 associated pulmonary aspergillosis. Mycoses.

[6] Koehler P, Bassetti M, Chakrabarti A (2021). Defining and managing COVID-19-associated pulmonary aspergillosis: the 2020 ECMM/ISHAM consensus criteria for research and clinical guidance. Lancet Infect Dis.

[7] Glass NL, Donaldson GC (1995). Development of primer sets designed for use with the PCR to amplify conserved genes from filamentous ascomycetes. Appl Environ Microbiol.

[8] Staab JF, Balajee SA, Marr KA (2009). Aspergillus section Fumigati typing by PCR-restriction fragment polymorphism. J Clin Microbiol.

[9] Bain JM, Tavanti A, Davidson AD, Jacobsen MD, Shaw D, Gow NA, Odds FC (2007). Multilocus sequence typing of the pathogenic fungus Aspergillus fumigatus. J Clin Microbiol.

[10] Russell CD, Fairfield CJ, Drake TM (2021). Co-infections, secondary infections, and antimicrobial use in patients hospitalised with COVID-19 during the first pandemic wave from the ISARIC WHO CCP-UK study: a multicentre, prospective cohort study. Lancet Microbe.

[11] Meersseman W, Vandecasteele SJ, Wilmer A, Verbeken E, Peetermans WE, Van Wijngaerden E (2004). Invasive aspergillosis in critically ill patients without malignancy. Am J Respir Crit Care Med.

[12] Su T, Li HC, Chen M (2009). Invasive pulmonary aspergillosis in patients with antineutrophil cytoplasmic antibody associated vasculitis. J Clin Rheumatol.

[13] Schauwvlieghe A, Rijnders BJA, Philips N (2018). Invasive aspergillosis in patients admitted to the intensive care unit with severe influenza: a retrospective cohort study. Lancet Respir Med.

[14] Bartoletti M, Pascale R, Cricca M (2021). Epidemiology of invasive pulmonary Aspergillosis among intubated patients with COVID-19: a prospective study. Clin Infect Dis.

[15] Fekkar A, Lampros A, Mayaux J (2021). Occurrence of invasive pulmonary fungal infections in patients with severe COVID-19 admitted to the ICU. Am J Respir Crit Care Med.

[16] RECOVERY Collaborative Group (2021). Tocilizumab in patients admitted to hospital with COVID-19 (RECOVERY): a randomised, controlled, open-label, platform trial. Lancet.

[17] Prattes J, Wauters J, Giacobbe DR (2022). Risk factors and outcome of pulmonary aspergillosis in critically ill coronavirus disease 2019 patients-a multinational observational study by the European Confederation of Medical Mycology. Clin Microbiol Infect.

[18] Chong WH, Neu KP (2021). Incidence, diagnosis and outcomes of COVID-19-associated pulmonary aspergillosis (CAPA): a systematic review. J Hosp Infect.

[19] Cornely OA, Maertens J, Bresnik M (2007). Liposomal amphotericin B as initial therapy for invasive mold infection: a randomized trial comparing a high-loading dose regimen with standard dosing (AmBiLoad trial). Clin Infect Dis.

[20] Hatzl S, Reisinger AC, Posch F (2021). Antifungal prophylaxis for prevention of COVID-19-associated pulmonary aspergillosis in critically ill patients: an observational study. Crit Care.

